# Most soil and litter arthropods are unidentifiable based on current DNA barcode reference libraries

**DOI:** 10.1093/cz/zoad051

**Published:** 2023-11-28

**Authors:** Ernesto Recuero, Frank E Etzler, Michael S Caterino

**Affiliations:** Department of Plant and Environmental Sciences, Clemson University, 277 Poole Agricultural Center, Clemson, SC 29634, USA; Department of Plant and Environmental Sciences, Clemson University, 277 Poole Agricultural Center, Clemson, SC 29634, USA; Natural Resource Section, Montana Department of Agriculture, 302 N Roberts St, Helena, MT 59601, USA; Department of Plant and Environmental Sciences, Clemson University, 277 Poole Agricultural Center, Clemson, SC 29634, USA

**Keywords:** Arthropoda, automated identification, DNA barcoding databases, taxonomy, taxonomic impediment

## Abstract

We are far from knowing all species living on the planet. Understanding biodiversity is demanding and requires time and expertise. Most groups are understudied given problems of identifying and delimiting species. DNA barcoding emerged to overcome some of the difficulties in identifying species. Its limitations derive from incomplete taxonomic knowledge and the lack of comprehensive DNA barcode libraries for so many taxonomic groups. Here, we evaluate how useful barcoding is for identifying arthropods from highly diverse leaf litter communities in the southern Appalachian Mountains (USA). We used 3 reference databases and several automated classification methods on a data set including several arthropod groups. Acari, Araneae, Collembola, Coleoptera, Diptera, and Hymenoptera were well represented, showing different performances across methods and databases. Spiders performed the best, with correct identification rates to species and genus levels of ~50% across databases. Springtails performed poorly, no barcodes were identified to species or genus. Other groups showed poor to mediocre performance, from around 3% (mites) to 20% (beetles) correctly identified barcodes to species, but also with some false identifications. In general, BOLD-based identification offered the best identification results but, in all cases except spiders, performance is poor, with less than a fifth of specimens correctly identified to genus or species. Our results indicate that the soil arthropod fauna is still insufficiently documented, with many species unrepresented in DNA barcode libraries. More effort toward integrative taxonomic characterization is needed to complete our reference libraries before we can rely on DNA barcoding as a universally applicable identification method.

Species are considered a fundamental unit in biology, and their characterization and delimitation have been of major importance in all fields of biology to study and understand Earth’s biodiversity and the complexity of biological processes (de Queiroz 2005). Nevertheless, we are far from knowing all species currently living on the planet, particularly in highly diverse areas in tropical regions and in such megadiverse groups as the Arthropoda. For instance, there are around one million named insect species currently recognized as valid, while estimates of the real number vary from around five million (Stork 2018) to as many as 30 million species (Erwin 1982). Other groups, even if not as diverse as insects, are even less known. Such is the case of mites, with roughly 50,000 recognized species, and estimates of real diversity above one million (Stork 2018). But even knowing and studying the already described species is a major task that requires considerable time and expertise as most of these groups lack specialized taxonomists able to identify and delimit species (Engel et al. 2021). This, in turn, increases the biases of study toward some groups, while others suffer further neglect.

By contrast, vertebrate species are often well-delimited and easily identified, even in the field, with only basic training. This has allowed the gathering of an enormous amount of information about many of these species, including distribution, year-to-year variation in abundance, phenology, ethology, biological interactions, etc. As a result, they have been used to propose general patterns in fields such as evolutionary biology or biogeography, and they are often the main protagonists in the study of biodiversity and its conservation (Titley et al. 2017). However, vertebrates represent an extremely small fraction of the total diversity on the planet, while our knowledge of organisms from highly diverse groups is in many cases reduced to the mere acknowledgment of their existence.

In trying to overcome the difficulty of identifying species, new methods have been developed over time. One of the most popular is DNA barcoding (Hebert et al. 2003). This method is based on the association of DNA sequences or barcodes with a particular species. In the case of Metazoa, Cytochrome Oxidase subunit I (Cox1) is the most widely used marker for generating taxonomically classified databases meant to allow comparison and subsequent identification of newly generated sequences (Andújar et al. 2018). DNA barcoding presents several advantages when trying to identify species. It requires little training, it can allow the identification of cryptic species, it can be used with a metabarcoding approach to process large number of samples or environmental DNA, it can help identify species from parts used as a food source, and it should help understanding the patterns of morphological variation within and among species (e.g., Joly et al. 2014; Purty and Chatterjee 2016; Pollack et al. 2018; DeSalle and Goldstein 2019). Additionally, the use of informatics and online resources allows the processing of large datasets in very short times. Most of these methods have been developed to analyze metabarcoding data from microbial communities but have also been proven useful in the study of other organisms (Porter and Hajibabaei 2018; Leray et al. 2022).

Barcoding also has limitations, particularly deriving from the incomplete taxonomic knowledge of the real diversity for most groups and in most geographic regions of the world. Most species are still to be discovered, characterized, formally described, and named. Even most of those that are already described are understudied, so databases are still incomplete and contain errors (Kwong et al. 2012; Meiklejohn et al. 2019), resulting in a barcoding taxonomic shortfall. The usefulness of DNA barcoding for species identification relies on the availability of complete databases including correctly identified barcodes, which can be used as a reference allowing an accurate assignment to species or at least some other supraspecific taxonomic rank (Moritz and Cicero 2004). Even if the two main reference databases, GenBank (Benson et al. 2013) and the Barcode of Life Data Systems (BOLD) (Ratnasingham and Hebert 2007), share data, they are each independent and present data that may be exclusive to one of them, so differences in results using one or the other are expected (e.g., Meiklejohn et al. 2019; Baena-Bejarano et al. 2023).

Global reference databases are also biased taxonomically and geographically. For instance, over 75% of species in the class Aves are represented in the BOLD database, and 73% of European Lepidoptera have been barcoded (Lopez-Vaamonde et al. 2021). On the other hand, representation in the BOLD database for the highly diverse order Coleoptera falls to around 12% of currently described species, which is just a fraction of the real number of its extant species on Earth. Additionally, most of these data are aggregated geographically, with much higher coverage in countries where there are active research programs to characterize biodiversity and complete DNA barcoding databases, such as in Canada, Germany, or Costa Rica (Ratnasingham and Hebert 2007; Geiger et al. 2016; Janzen and Hallwachs 2019). In this sense, it is expected that using DNA barcoding to identify species will perform differently depending on the database used, the taxonomic group, and the geographic region where the data come from.

Here, we evaluate the performance of DNA barcoding for species identification for litter and soil arthropods from the southern Appalachians in eastern North America, a relatively well-studied geographic region, by using 3 different databases and several automatic classification methods. We hypothesize that classification success at the species level in poorly studied taxa will be low, that performance among the different studied groups will be different, and that classification at higher levels such as genus or family should result in a higher number of correctly assigned taxa.

## Material and Methods

### Sampling

**Figure 1.**
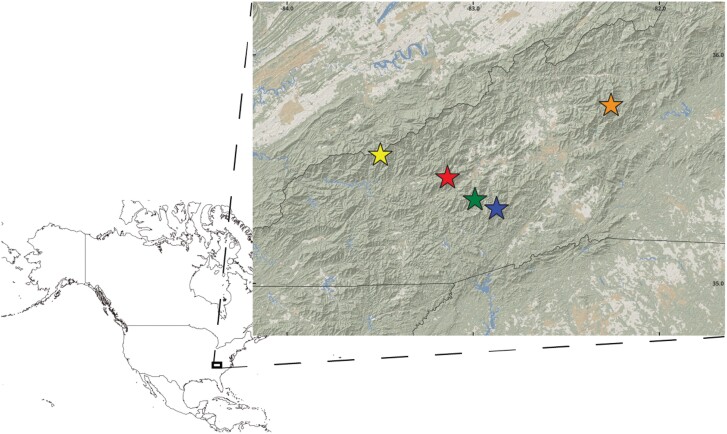
Sampling localities in the Southern Appalachian Mountains. Yellow star: Clingmans Dome; red star: Browning Knob; green star: Richland Balsam; blue star: Black Balsam Knob; orange star: Big Tom.

Soil arthropod samples were collected from 5 different high elevation sites located around 1,850–2,000 m in the mountains of southern Appalachia (Figure 1): Big Tom (35.7797°N 82.2599°W), Black Balsam Knob (35.328°N 82.8746°W), Browning Knob (35.4641°N 83.1378°W), Clingmans Dome (35.5629°N 83.4986°W), and Richland Balsam (35.3676°N 82.9903°W). Most of these localities represent sky islands characterized by the presence of coniferous forests dominated by *Picea rubens* and *Abies fraseri*, a kind of habitat restricted to the highest portions of the Appalachians from southwestern Virginia to western North Carolina and eastern Tennessee. At Black Balsam Knob the forest has been largely replaced by grassland.

At each locality, 3 independent samples of approximately 1 m^2^ forest floor litter were taken and sifted using an 8 mm mesh screen sifter.

Each litter sample was stored and transported to the laboratory in nylon bags. Arthropod samples were extracted from the litter using Berlese funnels and collected and preserved in 100% ethanol, storing them at −20 °C. Samples were initially sorted to order level, then they were sorted to unique morphotypes to which we refer as morphospecies. One specimen per morphospecies and per site was selected for DNA extraction and barcode sequencing. These specimens were digitally imaged before dissection; voucher pictures, in dorsal or lateral view, were taken with a Canon 6D SLR equipped with a 65-mm MP-E 1–5 × macro lens, using Visionary Digital Passport; we took 10 images per specimen that were stacked using Helicon Focus v5.3 (http://www.heliconsoft.com). Images are available at: https://www.flickr.com/photos/183480085@N02/

### DNA extraction, sequencing, and sequence processing

For most samples whole specimens were punctured and used for DNA extraction; if specimens were too large, we used legs or heads as tissue. Extractions were performed in 96-well plates using a magnetic-bead-based method with the Mag-Bind^®^ Blood & Tissue DNA HDQ 96 Kit (Omega Bio-tek, Inc.), following the manufacturer’s protocol, and a Hamilton Microlab^®^ Star robotic liquid handler system. Specimens were digested for a minimum of 2 h and, when possible, the remaining parts of the vouchers were recovered and stored in 70% ethanol and processed to be deposited in the Clemson University Arthropod Collection (http://www.cuacinsects.org/). After digestion the DNA in the digestion buffer was stored for a few days at −20 °C until purification was completed.

A 421 bp fragment of the Cytochrome Oxidase subunit I mitochondrial gene (COI), sitting within the conventional barcoding region, was selected and amplified via polymerase chain reaction (PCR) using primers BF2 and BR2 (Elbrecht and Leese 2017). We used indexed primers, including a unique combination of 9-bp tags in forward and reverse primers in order to identify each barcode after high-throughput sequencing (Meier et al. 2016). PCR reactions were performed in a total volume of 12.5 μL including 0.25 units of Platinum Taq DNA polymerase (Invitrogen), 15 pmol of each primer, 2.5 nmol of dNTPs, 19 nmol of MgCl_2_, 1.25 μL of magnesium-free 10× PCR buffer (Invitrogen) and 1 μL of template. PCR conditions consisted in an initial denaturation at 95 °C for 5 min followed by 35 cycles of denaturing (95 °C, 30 s), annealing (50 °C, 30 s), and extension (72 °C, 30 s), and a final extension step at 72 °C for 5 min.

For sequencing, 2 μL aliquots of each PCR product were combined into a single library mix. Four 100 μL aliquots of the combined PCR library were purified using the Mag-Bind Total Pure NGS kit (Omega Bio Tek), following the manufacturer protocol and using a 0.7× bead ratio to eliminate DNA fragments smaller than our target; once cleaned they were combined. Purified library concentration was measured using Qubit High Sensitivity assay and prepared for sequencing on an Illumina MiSeq using the MiSeq Reagent Kit v3 (Illumina) following the manufacturer’s protocol, including 15% of phiX control reads.

Illumina reads were processed with bbtools software package (https://jgi.doe.gov/data-and-tools/bbtools/%20) to merge paired-end reads, remove PhiX reads, trim Illumina adapters, filter reads for the correct size, remove reads with quality score < 30, cluster sequences by similarity allowing 5 mismatches (~1%) and generate a final matrix in FASTA format. Sequences were aligned with the online version of Mafft v7 (Katoh et al. 2019) using the auto strategy.

### Automated species identification

In order to assign a taxonomic identity to our recovered barcodes we used 3 different databases, MIDORI Reference 2 for COI as implemented in MIDORI Server (Leray et al. 2018, 2022), the Eukaryote CO1 Reference Set v4.0.1 (Porter and Hajibabaei 2018; available at https://github.com/terrimporter/CO1Classifier), which was analyzed using the Ribosomal Database Project (RDP) classifier (Wang et al. 2007), and the BOLD database through the BOLD Identification System for COI (IDS; available at http://www.boldsystems.org/index.php/IDS_OpenIdEngine).

MIDORI Reference 2 for COI is a matrix including all Eukaryotes, directly based on GenBank entries including precise and definite taxonomic information at species level (Machida et al. 2017). The MIDORI server (available at http://www.reference-midori.info/server.php) implements 3 different classification methods: RDP Classifier, Spingo (Allard et al. 2015) and Sintax (Edgar 2016). RDP and Sintax provide classifications at several taxonomic levels, from superkingdom to species, and a probability value for its classifications. Spingo provides classification at genus and species level, also providing a probability value. We selected the Unique COI database, which contains all unique haplotypes from all included species, and used default parameter values for all 3 methods.

RDP classifier was initially developed for the taxonomic assignment of bacterial rRNA barcodes, but it has proven to be an efficient classifier for other groups and genes, provided that an adequate reference database exists. The Eukaryote COI Reference Set v4.0.1, is a database specifically curated to classify COI barcodes of Eukaryote species, with particular emphasis on Arthropoda and Vertebrata (Porter and Hajibabaei 2018). As in MIDORI Reference, this database is based on GenBank entries with accurate taxonomic information at the species level, including multiple sequences per species if available and excluding fragments smaller than 500 bp.

When using methods that provide probability values, we considered an assignment correct when the proposed taxonomic identification was either based on multiple positive matches to independent barcodes in the used databases or when it matched our own identifications, and the probability value was 0.8 or higher. If the proposed taxonomic ID did not match the previous criteria, and the probability was 0.8 or higher, then we classified it as a false positive. We considered a barcode unidentified when the probability was lower than 0.8, even if in some cases the taxonomic assignment was correct (false negative). Taxonomic identifications were confirmed or rejected by direct morphological examination by the authors and collaborators (see Acknowledgments) and identification of the vouchers to the lowest taxonomic level we could achieve, most frequently at family level (or at least superfamily, but sometimes to tribe) (35.5% of all samples) but also to genus (28.1%), species (26.9%) or order (9.5%) (Supplementary Table S1). Some final identifications were refined to lower taxonomic levels when adequate barcode information was available (i.e., confirming preliminary morphological identifications, multiple good matches with sequences from independent sources, and/or good matches with sequences from papers authored by a specialist in the given taxon).

The BOLD Identification System for COI returns, when one is possible, a species-level identification including a probability of placement. For this, we used the Species Level Barcode Records Database, which includes all BOLD COI barcodes with a length of 500 bp or longer and a taxonomic identity at the species level (including also sequences with interim classification). The largest inconvenience for this approach is that, if there are no matches at the species level, no higher taxonomic level classification is suggested. Using the database including all barcode records on BOLD the search engine returns a list of the 20 top matches for each analyzed sequence, with their percentage of similarity but with no probability of placement to a taxon. In many cases, browsing this list of results provides useful information at the family or even genus levels, similar to a GenBank Blast search.

## Results

A total of 487 identifiable amplicons were successfully sequenced out of the 751 originally included samples, representing a 65.4% sequencing success (GenBank accession numbers are included in Supplementary Table S1). Samples that failed to yield sequence could be the result of PCR failure, caused by either very low DNA concentrations after extraction, presence of inhibitors in some extractions, or primer mismatch for some of the species included. Also, some barcodes may be amplified with low final amplicon concentrations, and they can become so diluted in the final library as to be missing during the sequencing process with high-throughput methods. We observed different sequencing success rates for the groups represented in our samples: Acari (146 amplicons out of 278 samples, 57% success), Araneae (43/54, 79.6%), Collembola (89/107, 83.2%), Coleoptera (147/173, 85.0%), Diptera (55/89, 61.8%), Hymenoptera (12/45, 24.4%), Protura (2/5, 40%), Pseudoscorpiones (3/5, 60%), Symphyla (2/2, 100%), and Diplopoda (1/1, 100%).

**Figure 2.**
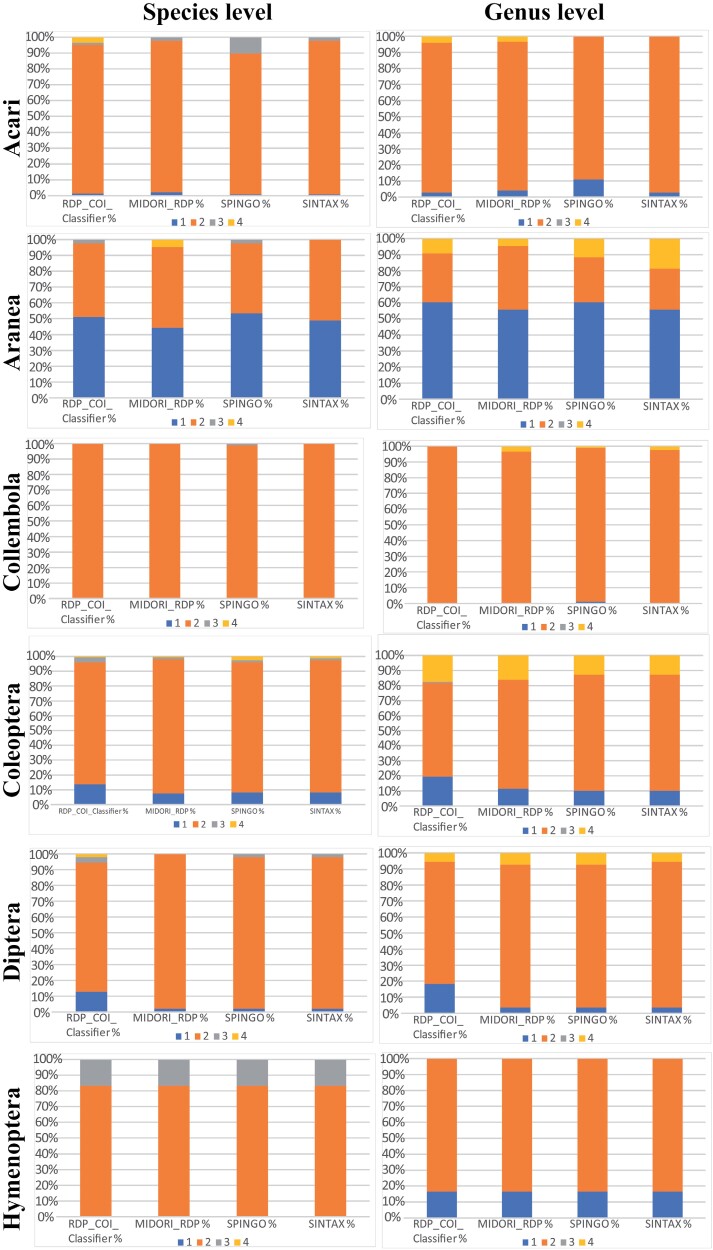
Success of automated identification (%) for the main taxonomic groups included in the analyses using different databases and informatics methods. Blue: correct identifications; orange: unidentified; gray: incorrectly identified (false positives); yellow: correctly identified with low probability (false negatives).

The taxonomic classification obtained using all databases yielded, in general, similarly poor results (Supplementary Table S1), but we observed differences between major taxa (Figure 2).

### Acari (Orders Mesostigmata, Oribatida, and Trombidiformes)

Classification of mites offered very poor results using both databases and all methods. Most barcodes could not be assigned to any taxon with high probability (>0.8). Out of the 146 barcodes examined, only 1 (0.7%, with the methods Spingo and Sintax), 2 (1.4%, RDP with COI Reference), or 3 (2%, RDP with MIDORI reference) were correctly assigned at the species level. In all 4 trials, we observed some false positives: 2 (1.4%, RDP with COI Reference), 3 (2%, RDP with MIDORI reference and Sintax), and 15 (10.2%, Spingo). However, in these cases, confirmed with individual blast searches in GenBank, the percent identities ranged from 90% to 94% with the suggested species, and they could in fact represent intraspecific variation, in which case the classification would be correct. In fact, at the genus level, there were no false positives, and we observed 4 (2.7%, RDP with COI Reference and Sintax), 6 (4.1%, RDP with MIDORI reference), and 16 (10.9%, Spingo) correctly identified barcodes. Some barcodes were correctly assigned by RDP analyses but showed low probabilities and thus were considered as false negatives, 6 at the species level using the COI Reference and 6 and 5 at genus level using the COI and the MIDORI references, respectively. Using BOLD IDS, the results for species identification was also very low, with only 5 barcodes correctly identified (3.4%). At higher taxonomic levels the classification obtained with MIDORI and COI Reference databases also showed very poor results. For instance, at the family level the only correct classifications corresponded to those barcodes correctly assigned at genus level. Additionally, only 5 (COI Reference), 6 (MIDORI reference) and 11 (Sintax) barcodes were correctly assigned to the right Order, while at the class level we observed the same number of correctly assigned barcodes, but up to 13 (COI Reference), 11 (Sintax) and 20 (MIDORI reference) false positives where Acari barcodes were identified as Insecta.

### Araneae

In the case of spiders, the success of the taxonomic automatic classification, even if low, was considerably better than for other groups. Around half of the barcodes were correctly identified at the species level: 19 (44.2%, RDP with MIDORI Reference), 21 (48.8%, Sintax), 22 (51.2%, RDP with COI Reference), and 23 (53.5%, Spingo). False positives were very rare, only 1 with RDP with COI Reference and with Spingo. False negatives were also rare, only 2 with RDP with MIDORI Reference. Classification at the genus level was even better, including 24 (55.8%, RDP with MIDORI Reference and Sintax) and 26 (60.5%, RDP with COI Reference and Spingo) correctly identified barcodes and no false positives. The number of false negatives remained relatively low, 2 with RDP with MIDORI Reference, 4 with RDP with COI Reference, 5 with Spingo, and 8 with Sintax. The classification using BOLD IDS offered slightly better results than the other methods, with correct species identification of 25 barcodes (56.8%). At higher taxonomic levels the proposed classifications are mostly correct, with all sequences correctly assigned at Order level and only 1 (RDP with MIDORI Reference and Sintax) or 2 (RDP with COI Reference) barcodes not identified at the Family level, although some of the correctly identified ones had low probabilities.

### Collembola (Orders Symphypleona, Entomobryomorpha and Poduromorpha)

In the case of Collembola, we observed the worst performance in all tried classifiers. No barcodes could be correctly assigned to species or genus using any of the methods or databases, including BOLD IDS; even at higher levels, up to class, we could not find any correct classification with a probability of 0.8 or higher. Only one barcode, using Spingo, was correctly assigned to the genus *Uzelia*. This sample is considered a false positive at the species level (GenBank blast percent identity of 90.95% with *Uzelia hansoni* sequences) but, considering the high intraspecific distances often observed in springtails (Porco et al. 2014), its classification could be correct. No other false positives were detected below the Class level, where barcodes were misidentified as Insecta with all methods.

### Coleoptera

Performance among Coleoptera barcodes was low. At the species level only 11 (7.5%, RDP with MIDORI reference), 12 (8.2%, Spingo and Sintax), and 20 (13.6%, RDP with COI Reference) barcodes were correctly assigned. The number of false positives was very low, with only 2 (RDP with COI Reference, Spingo and Sintax) or 5 (RDP with COI Reference) barcodes incorrectly identified, and usually associated with very closely related taxa with low interspecific genetic distances. Performance at the genus level was not much better, with 15 (10.2%, Spingo and Sintax), 17 (11.6%, RDP with MIDORI reference), and 29 barcodes correctly assigned (19.7%, RDP with COI Reference), and only one false positive observed with RDP with COI Reference. Classifications using BOLD IDS yielded 28 correct barcode identifications (21.6%), but also 5 false positives at species level, all of them belonging to closely related species of the genus *Geostiba* Thomson, 1858, not present in the database. Another false positive, a *Lasioderma serricornis* (Fabricius, 1792) barcode initially identified by BOLD as *Stegobium paniceum* (Linnaeus, 1758), was caused by the presence of a misidentified sequence in BOLD, but this was corrected a few weeks after it was observed. This case was surprising as, among the top 20 matches (all of them with a 100% similarity), 19 were correctly identified and only 1 was misidentified, so the method seems to ignore frequencies of reference barcodes. Correct classifications with high probability are also low at the family level; the best results, obtained from RDP with COI Reference, included 42 barcodes (28.6%) correctly identified to Family.

### Diptera

Classification of Diptera barcodes at the species level resulted in 1 (1.9%, RDP with MIDORI reference, Spingo and Sintax) to 6 (11.1%, RDP with COI reference) correctly identified barcodes. False positives were also low and observed only with RDP with COI reference (3 barcodes, 5.6%), RDP with Midori reference (1 barcode,1.9%) and Spingo and Sintax (2 barcodes, 3.7%). One of the false identifications resulted from barcodes initially identified as *Nearcticorpus pecki* Marshall & Roháček, 1982 but then reidentified as *N*. *canadense* Roháček & Marshall, 1982; this species-level classification is updated in the BOLD database but not in GenBank. At the genus level resolution was similar, with 2 (3.6%, RDP with MIDORI reference, Spingo and Sintax) to 9 (16.4%, RDP with COI reference) correct results. No false positives were observed for genera. With BOLD IDS we recovered 11 correctly identified barcodes (20%), with apparently no false positives. At higher taxonomic levels we observed no better results than with genera, with only 9 barcodes correctly assigned to Family and 14 to the order Diptera with high probabilities.

### Hymenoptera

Among Hymenoptera, only 2 barcodes (16.7%) corresponding to ants were correctly identified to genus and species as *Stenamma diecki* Emery, 1895 by all methods and both databases. The remaining barcodes were not identified, but no false positives were observed. No positive identifications were obtained using BOLD IDS. Similarly, at the Family level, only those 2 barcodes were correctly assigned to Formicidae. Among the other barcodes we observed very low probabilities, although members of the family Braconidae were identified as such and can be considered as false negatives.

### Underrepresented groups

Other small groups were poorly represented among our barcodes, including 1 Diplopoda, 2 Symphyla, 3 Pseudoscorpiones, and 2 Protura. No good classification was obtained at any taxonomic level for any of these barcodes, except for one pseudoscorpion that, in all trials but the RDP with COI Reference, was correctly assigned to the genus *Novobisium* Muchmore, 1967. However, its classification at the species level as *N*. *tenue* (Chamberlin, 1930) might be a false positive (GenBank Blast percent identity of 94.54%). We could not identify any of these barcodes using BOLD IDS.

## Discussion

After almost 2 decades of being formally defined, DNA barcoding remains a promising method to facilitate species identification that has proven successful in several cases (e.g., Lukhtanov et al. 2009; Kelly et al. 2011; Young et al. 2021; Baena-Bejarano et al. 2023), but there are many cases of poor performance under most circumstances (e.g., Meier et al. 2006; Jiang et al. 2014; Meierotto and Sikes, 2015; Gibbs 2018; Stallman et al. 2019).

**Figure 3.**
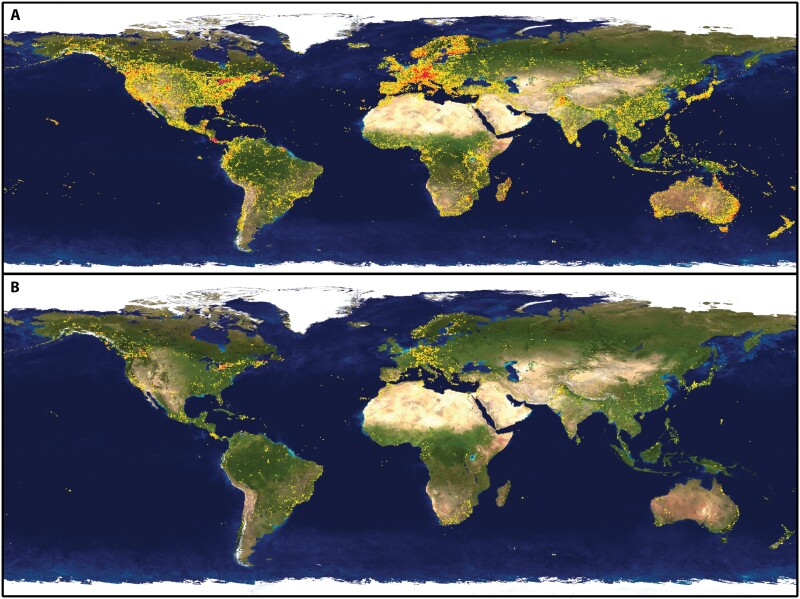
Global coverage of barcode records in BOLD database for (A) Insecta, and (B) Arachnida (https://boldsystems.org, accessed 7 May 2023).

**Figure 4.**
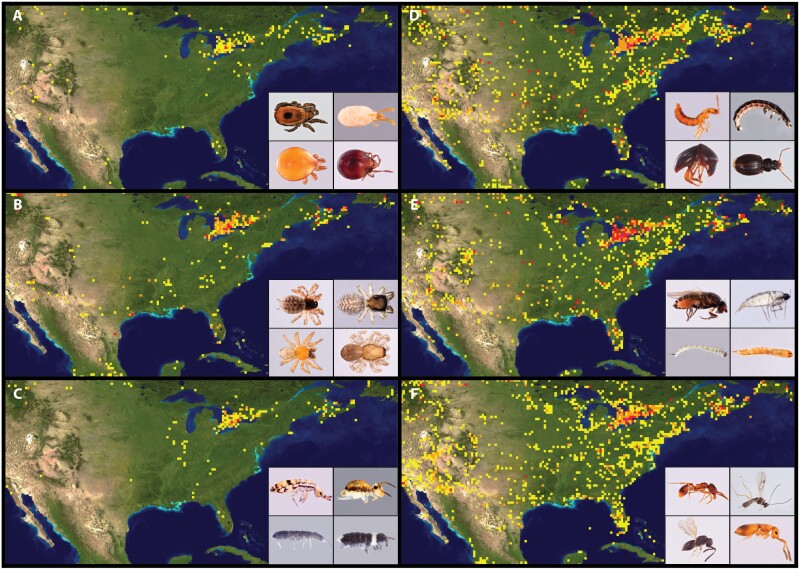
Barcode coverage in eastern North America for (A) Acari, (B) Araneae, (C) Collembola, (D) Coleoptera, (E) Diptera, and (F) Hymenoptera (https://boldsystems.org, accessed 7 May 2023).

In our case, focused on litter arthropods from the Southern Appalachian Mountains, the observed results indicate poor performance in all considered groups, although some of them performed better than others (Figure 2). This is caused mainly by the major drawback of using DNA barcoding for the identification of organisms, which is the lack of complete databases including correctly identified species and their intraspecific variability for a large portion of the species diversity across taxonomic groups and around the planet (Weigand et al. 2019; Eldred et al. 2021; Kolter and Gemeinholzer 2021). Two major public databases are the main source of reference sequences for barcoding, NCBI GenBank and BOLD. Even if both databases are independent, they share information; BOLD sequences are submitted to GenBank once they become public, while BOLD database periodically incorporates sequences from GenBank with complete taxonomic information, so it is expected that results using both databases should offer similar performance (Pentinsaari et al. 2020). Both databases are continually growing with contributions from researchers around the globe, but the number of represented species in many groups is far from complete. For instance, a search in the BOLD database shows a total of 249,438 identified species of Insecta with barcodes (accessed 18 July 2023). Considering that currently there are over one million recognized species, that number suggests that almost one fourth of them are represented in the database. However, if we consider conservative estimations of the real diversity of around 5 million insect species in the World (Stork 2018), then the representation reaches only about 5% of the total diversity of the class Insecta. Another diverse group of arthropods, Arachnida, is represented in BOLD database by 17,641 species with a name and a barcode (less than 20% of the current number of species) of which, for instance, 10,512 correspond to spiders (21% of current diversity) and 4,349 to mites (less than 10%). Considering estimates of real species numbers for these two arachnid groups (Stork 2018), representation would be about 12% and 0.5%, respectively. In the case of Collembola, the group with the poorest identification success, there are 1,383 species with barcodes, representing around 15% of currently named species. However, considering different estimates of real diversity, such a number would represent between 0.3% and 3% (Bellini et al. 2023). Additionally, geographic coverage of the represented species is uneven, with few moderately to highly prospected and studied areas, such as northeastern North America or Central Europe, and many large regions poorly sampled, especially in Asia, Africa, and South America (Figure 3). Differences at a regional scale are also quite pronounced (Figure 4).

It is somewhat surprising that even in a relatively well-studied geographic area like eastern Northern America, there is still such a large taxonomic shortfall of barcode data for some of the most relevant taxonomic groups in the soil communities. The heterogeneity in representation between taxonomic groups is probably the main reason for the observed differences in barcode identification success (Virgilio et al. 2010), and this heterogeneity evidently extends to within major taxa as well, with small-bodied members in neglected microhabitats proportionally underrepresented. In general, available reference databases and methods are not useful to identify most soil arthropods from the southern Appalachians. Only spiders yield a moderate rate of success, allowing the identification of around half of the barcodes and with low numbers of false positives. It is interesting that, although the geographic distribution of available spider reference barcodes is limited, there is a concentration of data from the Southern Appalachians (Figure 4B).

In other groups, like springtails, failure was almost absolute, and in mites, the number of correctly identified species is similar to the number of false positives. Both groups have very limited representation of reference barcoding data from the Southern Appalachians (Figure 4A,C). Even at higher taxonomic ranks, automatic classifiers performed poorly, and surprisingly, very few barcodes were correctly identified. Mites, probably one of the most diverse but more neglected groups of soil arthropods, were mostly correctly identified at least to family and order level by Young et al. (2021). However, they were working with species from Canada, a country well characterized for barcoding studies (Figure 4A), which again is indicative that the success of identification using barcoding is biased not only regarding taxonomic groups but also geographic regions. Still, one might have guessed that higher level taxonomic differences between regions of eastern North American would not have been so great as to preclude high level identification in Appalachia. It could be that saturation of Cox1 sequences, caused by very old ages of certain clades, could add up to a low representation in databases as to prevent a correct assignment to family or, in some cases, even to order. Also, classic supraspecific taxa often have a subjective component and groups with the same taxonomic rank may have very diverse ages; this can affect, for instance, the setting of genetic distance thresholds to automatically identify a barcode to higher taxonomic level.

In general, we have observed slightly better performance when using BOLD as the reference database. A few studies compared performance of both databases when identifying species of different organisms. For instance, Meiklejohn et al. (2019) found that GenBank performed better than BOLD identifying insect species with a small number of barcodes, while results were similar among databases for plants and macro-fungi. However, a revision of the insect sequences used in that study showed that the differences were not real (Pentinsaari et al. 2020). In fact, analysis of a much larger insect dataset from Colombia suggests that in general BOLD outperforms GenBank, particularly in groups such as Coleoptera, identifying a large number of barcodes to family, genus and even species (Baena-Bejarano et al. 2023). Among methods used here we found no large differences, and performance varied across methods depending on the studied group, as had been previously observed (Leray et al. 2022). Given this variance in the number of species identified, and the short times taken to produce the identifications, we recommend using multiple methods to maximize the number of identified barcodes from a given dataset.

The generation of barcodes can have its own problems, as we have observed with our own results; for instance, we observed different rates of sequencing success between the different studied groups, with high success in Coleoptera and Collembola but high levels of failure in other groups, particularly in Hymenoptera. The development of next-generation sequencing techniques allows the generation of huge amounts of sequences. Doing this can reduce costs per barcode both in terms of time and money (Shokralla et al. 2014; Srivathsan et al. 2021), but to make this effective it is necessary to optimize general conditions, sometimes across very different organisms. However, the generalization of procedures and conditions can influence the final results. It is likely that at least a large portion of the observed differences in our data can be explained by a poor affinity of the used primers, although problems sequencing hymenopteran barcodes have been observed in other works, particularly in some families such as Braconidae or Formicidae, and they may require special conditions (Sint et al. 2014). In these cases, it could be a good idea to split amplification work into different taxonomic groups so more specific primers and reaction conditions can be used. Also, if the available resources allow it, it would be a good idea to include several specimens per species to overcome potential failures during DNA extraction. Also, targeting smaller fragments can increase amplification success, particularly when working with degraded DNA (e.g., dried museum specimens), although a minimum size is required by databases as BOLD (fragments larger than 500 bp) to meet compliance criteria (Hubert et al. 2008). The size of the barcode fragment can have an effect when characterizing community diversity (Huber et al. 2009), and it could also affect species identification, especially when analyzing closely related species with low genetic divergences. However, barcode fragments as small as 135 bp have proven to be informative (Hajibabaei et al. 2006; Yeo et al. 2020); at this point the 500 bp criteria seems arbitrary and it could be beneficial to incorporate also smaller barcodes into global barcoding databases to increase taxonomic coverage. In our case, considering the lack of resolution even above the species level, we do not consider that barcode fragment size is a major factor affecting the observed overall results.

Our results show worse performance in identifying barcodes at low taxonomic levels than other studies dealing with insects from different parts of the world (e.g., Nneji et al. 2020; Pentinsaari et al. 2020; Baena-Bejarano et al. 2023). However, none of those studies was centered on the soil fauna, indicating that there may be another bias in databases associated with ecological factors. Indeed, soil communities represent a huge reservoir of biodiversity still poorly documented, with many undescribed taxa even in relatively well-studied regions (André et al. 1994; Briones 2014). Traditionally neglected groups in biological science, as is the case for so many arthropod taxa, are difficult to study, in part because they can be taxonomically complex and there are very few specialists trying to cover a large diversity of species. Identifying species in such groups is beyond the skills of most zoologists, so the numbers of studies considering them at the species, or even genus level, are limited. More commonly, large-scale community studies generalize about them at the family or even order level, considering that they represent single functional groups. Or they are simply ignored entirely. However, they represent outstanding elements in every ecosystem, adding large parts of the total diversity and even biomass, and likely playing diverse key roles in the ecological networks.

Improving our ability to identify species from such poorly known groups would certainly facilitate their study, resulting in more data and the possibility to consider larger portions of real biodiversity when interpreting the complexity of biological processes, both at local and global scales. Also, from an applied perspective, identification of frequently overlooked species can be of major importance for example to prevent or predict the expansion of diseases, identify threats caused by invasive organisms, detect and control agricultural and forest pests, develop conservation plans to preserve natural areas and their ecological processes, etc. (e.g., Dehling 2018; Suffert et al. 2018; Bezeng and van der Bank 2019; Brugueras et al. 2020). At this point, we have a methodology that works, but curiously we lack the basic knowledge to make it work properly. When designing a project using DNA barcoding identifications, for instance inventorying species diversity at a given place, it remains hard to predict how well this method will perform, as databases are biased geographically, taxonomically, and ecologically. To improve and complete our reference databases, both locally and globally, it is still extremely necessary to invest in setting deep foundations for our knowledge of diversity, by funding and promoting the development of integrative taxonomic research, training students in taxonomic practices, and supporting the invaluable labor of scientific collections.

## Supplementary Material

Supplementary material can be found at https://academic.oup.com/cz.

zoad051_suppl_Supplementary_Table_S1
